# *catena*-Poly[[[diaqua­(tetra­methyl­ethylenediamine-κ^2^ *N*,*N*′)nickel(II)]-μ-sulfato-κ^2^ *O*:*O*′] monohydrate]

**DOI:** 10.1107/S1600536813006557

**Published:** 2013-03-13

**Authors:** Guntram Schmidt, Kurt Merzweiler

**Affiliations:** a Institut für Chemie, Naturwissenschaftliche Fakultät II, Martin-Luther-Universität Halle-Wittenberg, Kurt-Mothes-Strasse 2, 06120 Halle (Saale), Germany

## Abstract

The title compound, {[Ni(SO_4_)(C_6_H_16_N_2_)(H_2_O)_2_]·H_2_O}_*n*_, contains a Ni^II^ atom that is coordinated nearly octa­hedrally by a chelating tetra­ethyl­enediamine (tmeda) ligand, two water mol­ecules in a *cis* arrangement and two O atoms of two sulfate anions in a *trans* arrangement. The sulfate anions act as μ_2_-bridging ligands leading to a chain structure of alternating NiO_4_N_2_ octa­hedra and SO_4_ tetra­hedra parallel to [001]. The polymeric chains are linked by O—H⋯O hydrogen bonds between coordinating water mol­ecules and sulfate anions to give double strands. There is a lattice water mol­ecule which is also involved in O—H⋯O hydrogen bonding between adjacent [Ni(SO_4_)(tmeda)(H_2_O)_2_] chains.

## Related literature

For crystal structures of oligo- and polymeric nickel(II) tmeda complexes, see: Anderson *et al.* (2009 ▶); Erer *et al.* (2010 ▶). For related literature on one-dimensional metal sulfates, see: Behera & Rao (2006 ▶).
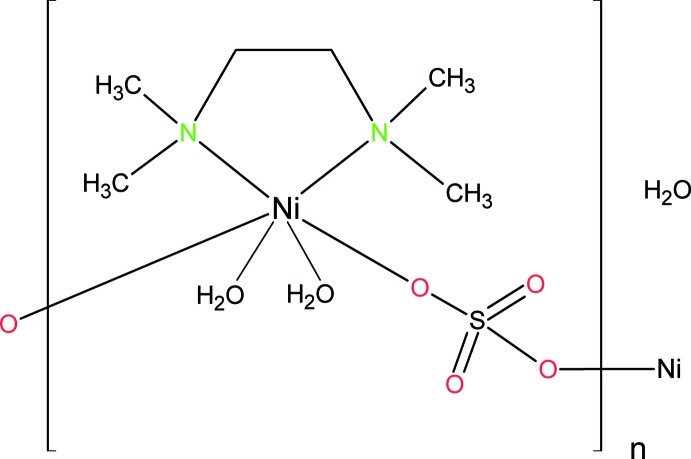


## Experimental

### 

#### Crystal data

[Ni(SO_4_)(C_6_H_16_N_2_)(H_2_O)_2_]·H_2_O
*M* *_r_* = 325.03
Orthorhombic, 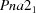

*a* = 21.108 (4) Å
*b* = 9.9335 (19) Å
*c* = 6.3879 (13) Å
*V* = 1339.4 (5) Å^3^
*Z* = 4
Mo *K*α radiation
μ = 1.63 mm^−1^
*T* = 223 K
0.48 × 0.11 × 0.11 mm

#### Data collection

Stoe IPDS diffractometer
Absorption correction: numerical (*IPDS*; Stoe & Cie, 1999 ▶) *T*_min_ = 0.648, *T*_max_ = 0.841
10054 measured reflections
2585 independent reflections
2291 reflections with *I* > 2σ(*I*)
*R*_int_ = 0.054

#### Refinement

*R*[*F* ^2^ > 2σ(*F* ^2^)] = 0.023
*wR*(*F* ^2^) = 0.052
*S* = 1.04
2585 reflections
176 parameters
7 restraints
H atoms treated by a mixture of independent and constrained refinement
Δρ_max_ = 0.37 e Å^−3^
Δρ_min_ = −0.31 e Å^−3^
Absolute structure: Flack (1983 ▶), 1167 Friedel pairs
Flack parameter: −0.005 (13)

### 

Data collection: *IPDS* (Stoe & Cie, 1999 ▶); cell refinement: *IPDS*; data reduction: *IPDS*; program(s) used to solve structure: *SHELXS97* (Sheldrick, 2008 ▶); program(s) used to refine structure: *SHELXL97* (Sheldrick, 2008 ▶); molecular graphics: *DIAMOND* (Brandenburg, 2009 ▶); software used to prepare material for publication: *SHELXL97*.

## Supplementary Material

Crystal structure: contains datablock(s) I, global. DOI: 10.1107/S1600536813006557/wm2722sup1.cif

Structure factors: contains datablock(s) I. DOI: 10.1107/S1600536813006557/wm2722Isup2.hkl

Additional supplementary materials:  crystallographic information; 3D view; checkCIF report

## Figures and Tables

**Table 1:** Hydrogen-bond geometry (Å, °)

*D*—H⋯*A*	*D*—H	H⋯*A*	*D*⋯*A*	*D*—H⋯*A*
O5—H2⋯O4^i^	0.84 (2)	1.84 (2)	2.672 (2)	171 (3)
O5—H1⋯O7	0.83 (2)	1.93 (2)	2.765 (3)	176 (3)
O6—H4⋯O3	0.83 (2)	1.89 (2)	2.687 (3)	160 (3)
O6—H3⋯O3^ii^	0.83 (2)	2.03 (2)	2.829 (3)	162 (3)
O7—H5⋯O4	0.83 (2)	2.12 (2)	2.899 (3)	157 (3)
O7—H6⋯O3^ii^	0.83 (2)	2.18 (2)	2.934 (3)	151 (3)

Symmetry codes: (i) 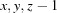; (ii) 
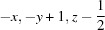
.

**Fig. 1.**
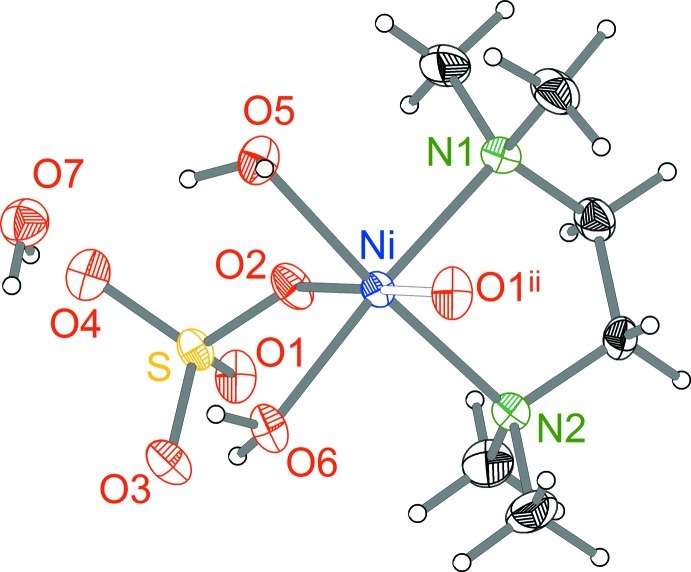
The coordination sphere around the nickel(II) atom in the structure of compound (I). The asymmetric unit is marked by solid lines of corresponding bonds. Displacement ellipsoids are drawn at the 50% probability level. [Symmetry code: ii) x, y, z-1].

**Fig. 2.**
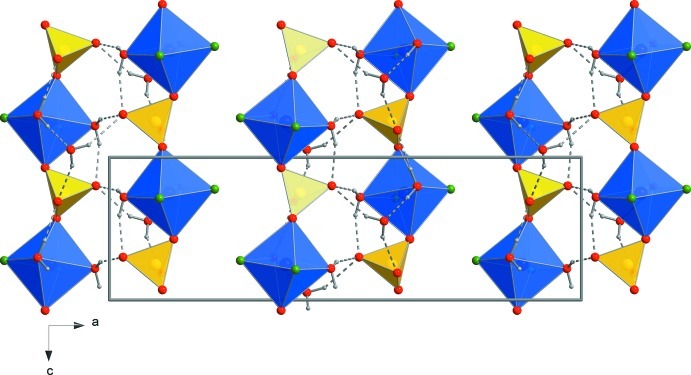
Polyhedral representation of the structure of compound (I). NiN2O4 octahedra are blue, SO4 tetrahedra are yellow.

**Fig. 3.**
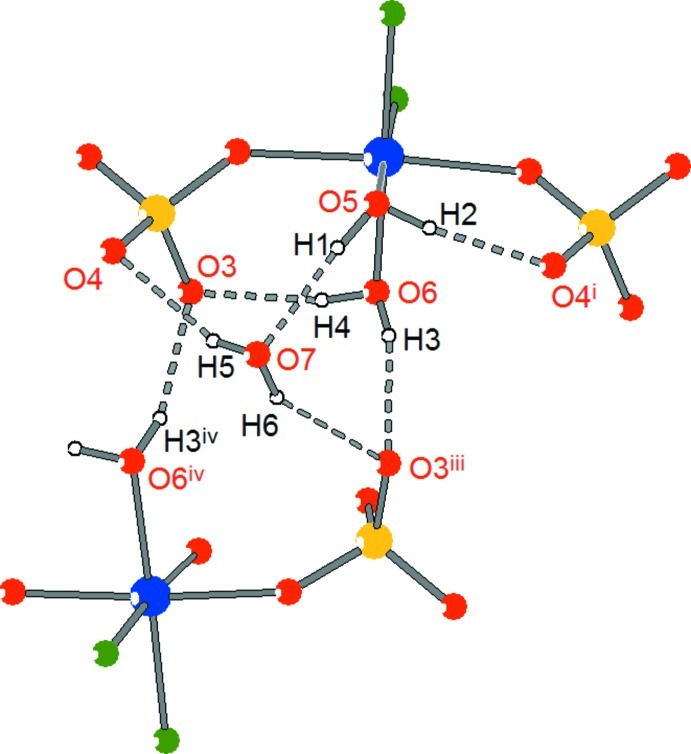
Part of the hydrogen-bonding network (dashed lines) in the structure of compound (I). [Symmetry codes: i) x, y, -z+1; iii) -x, -y+1, z-0.5; iv) -x, -y + 1, z + 0.5.]

## supplementary crystallographic information

### Comment

The title compound, {[Ni(H_2_O)_2_(C_6_H_16_N_2_)(SO_4_)]^.^H_2_O}_n_, (I), forms a coordination polymer which consists of an alternating arrangement of [Ni(H_2_O)_2_(tmeda)]^2+^ (tmeda is tetraethylenediamine) cations and SO_4_^2-^ anions. The coordination around nickel(II) is roughly octahedral with a chelating tmeda ligand, two water molecules in *cis* positions and two oxygen atoms of the SO_4_^2-^ anions in a *trans* arrangement (Fig. 1). In a polyhedral description the chain consists of corner sharing NiN_2_O_4_ octahedra and SO_4_ tetrahedra (Fig. 2). The observed Ni—N (2.141 (2), 2.1453 (19) Å) and Ni—O(aqua) distances (2.0639 (17), 2.084 (2) Å) agree well with the values reported for other nickel(II) complexes containing tmeda and aqua ligands, *e.g.* [Ni_6_(CO_3_)_4_(tmeda)_6_]Cl_4_^.^CH_2_Cl_2_ (Anderson *et al.*, 2009) and [Ni(C_4_O_4_)(tmeda)(H_2_O)_2_]^.^H_2_O (Erer *et al.*, 2010). A related chain structure consisting of corner-sharing NiO_4_(H_2_O)_2_ octahedra and SO_4_ tetrahedra has been observed in the compound [C_2_N_2_H_10_][Ni(SO_4_)_2_(H_2_O)_2_] (Behera & Rao, 2006). However, in contrast to compound (I) each NiO_4_(H_2_O)_2_ octahedron of the chain is connected to four SO_4_ tetrahedra.

The crystal packing of the [Ni(H_2_O)_2_(tmeda)]SO_4_ chains is supported by a network of hydrogen bridges (Figs. 2 and 3). Each of the water molecules attached to nickel forms an intrachain hydrogen bond of the type *R*^1^_1_(6) or *R*^1^_1_(6) to a sulfate oxygen atom. The lattice water molecule, which acts as a linker between the chains, forms two hydrogen bonds of the type *R*^1^_1_(2) to sulfate oxygen atoms and a *D*^1^_1_(2) hydrogen bond to a coordinating water molecule. Additonally, there are hydrogen bond motifs of the type *R*^2^_2_(12) which are formed between coordinating water molecules and sulfate oxygen atoms of neighbouring chains. As a result of these interchain hydrogen bridges, double strands are formed which propagate parallel to [001]. The arrangement of the double strands corresponds to a distorted hexagonal rod packing.

### Experimental

3.7 ml (46 mmol) of pyridine were added to a solution of 2.0 g (7.6 mmol) of nickel sulfate hexahydrate in 50 ml of water. 1.2 ml (8.0 mmol) of tmeda were added and all volatile compounds removed under reduced pressure at 333 K. The resulting solid was recrystallized from water and washed with ether to obtain the title compound in a yield of 85% (2.1 g).

### Refinement

The hydrogen atoms of the tmeda ligand were positioned geometrically and were refined using a riding model with *U*(H) = 1.2 *U*_eq_(C). Hydrogen atoms of the water molecules were located from difference Fourier maps and were refined with O—H distances fixed in the range of 0.83–0.84 Å and *U*(H) = 1.2*U*_eq_(O).

### Crystal data

**Table:**

[Ni(SO_4_)(C_6_H_16_N_2_)(H_2_O)_2_]·H_2_O	*F*(000) = 688
*M**_r_* = 325.03	*D*_x_ = 1.612 Mg m^−^^3^
Orthorhombic, *P**n**a*2_1_	Mo *K*α radiation, λ = 0.71073 Å
Hall symbol: P 2c -2n	Cell parameters from 8000 reflections
*a* = 21.108 (4) Å	θ = 2.2–25.8°
*b* = 9.9335 (19) Å	µ = 1.63 mm^−^^1^
*c* = 6.3879 (13) Å	*T* = 223 K
*V* = 1339.4 (5) Å^3^	Block, green
*Z* = 4	0.48 × 0.11 × 0.11 mm

### Data collection

**Table:**

Stoe IPDS diffractometer	2585 independent reflections
Radiation source: fine-focus sealed tube	2291 reflections with *I* > 2σ(*I*)
Graphite monochromator	*R*_int_ = 0.054
area detector scans	θ_max_ = 25.9°, θ_min_ = 2.3°
Absorption correction: numerical (*IPDS*; Stoe & Cie, 1999)	*h* = −25→25
*T*_min_ = 0.648, *T*_max_ = 0.841	*k* = −12→12
10054 measured reflections	*l* = −7→7

### Refinement

**Table:**

Refinement on *F*^2^	Secondary atom site location: difference Fourier map
Least-squares matrix: full	Hydrogen site location: inferred from neighbouring sites
*R*[*F*^2^ > 2σ(*F*^2^)] = 0.023	H atoms treated by a mixture of independent and constrained refinement
*wR*(*F*^2^) = 0.052	*w* = 1/[σ^2^(*F*_o_^2^) + (0.0286*P*)^2^] where *P* = (*F*_o_^2^ + 2*F*_c_^2^)/3
*S* = 1.04	(Δ/σ)_max_ = 0.001
2585 reflections	Δρ_max_ = 0.37 e Å^−^^3^
176 parameters	Δρ_min_ = −0.31 e Å^−^^3^
7 restraints	Absolute structure: Flack (1983), 1167 Friedel pairs
Primary atom site location: structure-invariant direct methods	Flack parameter: −0.005 (13)

### Special details

**Table:**

Geometry. All e.s.d.'s (except the e.s.d. in the dihedral angle between two l.s. planes) are estimated using the full covariance matrix. The cell e.s.d.'s are taken into account individually in the estimation of e.s.d.'s in distances, angles and torsion angles; correlations between e.s.d.'s in cell parameters are only used when they are defined by crystal symmetry. An approximate (isotropic) treatment of cell e.s.d.'s is used for estimating e.s.d.'s involving l.s. planes.
Refinement. Refinement of *F*^2^ against ALL reflections. The weighted *R*-factor *wR* and goodness of fit *S* are based on *F*^2^, conventional *R*-factors *R* are based on *F*, with *F* set to zero for negative *F*^2^. The threshold expression of *F*^2^ > σ(*F*^2^) is used only for calculating *R*-factors(gt) *etc*. and is not relevant to the choice of reflections for refinement. *R*-factors based on *F*^2^ are statistically about twice as large as those based on *F*, and *R*- factors based on ALL data will be even larger.

### Fractional atomic coordinates and isotropic or equivalent isotropic displacement parameters (Å^2^)

**Table:**

	*x*	*y*	*z*	*U*_iso_*/*U*_eq_	
C1	0.24703 (13)	0.7260 (3)	0.0085 (5)	0.0305 (6)	
H1C	0.2232	0.7845	−0.0842	0.037*	
H1B	0.2424	0.6334	−0.0371	0.037*	
H1A	0.2914	0.7511	0.0051	0.037*	
C4	0.16332 (11)	0.9525 (2)	0.2039 (4)	0.0224 (6)	
H4B	0.1637	1.0473	0.2462	0.027*	
H4A	0.1625	0.9489	0.0507	0.027*	
C2	0.26524 (13)	0.6637 (3)	0.3636 (5)	0.0329 (7)	
H2C	0.2506	0.6717	0.5070	0.039*	
H2B	0.3079	0.6996	0.3527	0.039*	
H2A	0.2653	0.5696	0.3229	0.039*	
C3	0.22243 (11)	0.8836 (3)	0.2836 (5)	0.0244 (6)	
H3B	0.2599	0.9279	0.2250	0.029*	
H3A	0.2244	0.8915	0.4364	0.029*	
C5	0.05032 (14)	0.9321 (3)	0.1665 (5)	0.0332 (7)	
H5C	0.0121	0.8923	0.2238	0.040*	
H5B	0.0552	0.9048	0.0216	0.040*	
H5A	0.0473	1.0294	0.1742	0.040*	
C6	0.09619 (15)	0.9283 (3)	0.5084 (5)	0.0316 (7)	
H6C	0.0580	0.8867	0.5626	0.038*	
H6B	0.0920	1.0254	0.5143	0.038*	
H6A	0.1322	0.9005	0.5922	0.038*	
N1	0.22264 (8)	0.7398 (2)	0.2241 (4)	0.0197 (5)	
N2	0.10595 (9)	0.8859 (2)	0.2890 (3)	0.0190 (5)	
Ni	0.125910 (11)	0.67593 (3)	0.25156 (6)	0.01456 (8)	
O1	0.11212 (10)	0.69565 (19)	0.9294 (3)	0.0245 (5)	
O2	0.13632 (9)	0.6444 (2)	0.5737 (3)	0.0232 (5)	
O3	0.02891 (8)	0.6195 (2)	0.7004 (3)	0.0262 (4)	
O4	0.10994 (9)	0.46409 (19)	0.8110 (3)	0.0265 (4)	
O5	0.15204 (9)	0.47588 (19)	0.2051 (3)	0.0240 (4)	
H1	0.1316 (12)	0.424 (3)	0.283 (4)	0.029*	
H2	0.1375 (14)	0.463 (3)	0.084 (3)	0.029*	
O6	0.03092 (8)	0.6302 (2)	0.2801 (3)	0.0234 (4)	
H3	0.0187 (12)	0.557 (2)	0.234 (6)	0.028*	
H4	0.0213 (14)	0.634 (3)	0.406 (3)	0.028*	
O7	0.08191 (10)	0.2998 (2)	0.4475 (3)	0.0305 (5)	
H5	0.0789 (16)	0.343 (3)	0.558 (4)	0.037*	
H6	0.0442 (10)	0.309 (3)	0.414 (5)	0.037*	
S	0.09690 (2)	0.60503 (5)	0.75426 (13)	0.01806 (12)	

### Atomic displacement parameters (Å^2^)

**Table:**

	*U*^11^	*U*^22^	*U*^33^	*U*^12^	*U*^13^	*U*^23^
C1	0.0277 (15)	0.0361 (17)	0.0278 (15)	−0.0013 (12)	0.0109 (12)	−0.0029 (14)
C4	0.0255 (13)	0.0152 (11)	0.0265 (17)	−0.0026 (9)	0.0032 (10)	0.0009 (11)
C2	0.0187 (13)	0.0386 (18)	0.0413 (17)	0.0035 (12)	−0.0036 (12)	0.0087 (16)
C3	0.0203 (11)	0.0238 (12)	0.0290 (17)	−0.0054 (9)	0.0000 (11)	−0.0024 (14)
C5	0.0262 (14)	0.0265 (15)	0.0470 (18)	0.0070 (12)	−0.0093 (12)	0.0022 (14)
C6	0.0396 (17)	0.0267 (16)	0.0284 (15)	0.0031 (12)	0.0099 (13)	−0.0099 (13)
N1	0.0173 (9)	0.0215 (10)	0.0202 (13)	0.0008 (7)	0.0012 (10)	−0.0005 (11)
N2	0.0178 (9)	0.0203 (10)	0.0188 (14)	−0.0005 (8)	0.0008 (8)	−0.0001 (10)
Ni	0.01562 (12)	0.01675 (14)	0.01132 (12)	−0.00151 (11)	−0.00008 (17)	0.00004 (18)
O1	0.0349 (11)	0.0235 (12)	0.0151 (10)	−0.0064 (8)	−0.0011 (9)	−0.0023 (9)
O2	0.0215 (10)	0.0346 (12)	0.0135 (9)	−0.0053 (8)	0.0024 (7)	0.0030 (9)
O3	0.0192 (8)	0.0360 (10)	0.0235 (11)	−0.0042 (7)	0.0023 (7)	0.0020 (8)
O4	0.0342 (10)	0.0240 (10)	0.0215 (11)	−0.0027 (8)	−0.0042 (7)	−0.0013 (8)
O5	0.0335 (10)	0.0216 (10)	0.0170 (11)	−0.0004 (8)	−0.0007 (7)	0.0005 (8)
O6	0.0223 (8)	0.0286 (9)	0.0193 (11)	−0.0084 (6)	0.0004 (9)	0.0028 (11)
O7	0.0331 (11)	0.0285 (12)	0.0298 (11)	0.0014 (9)	0.0020 (9)	−0.0013 (10)
S	0.0190 (2)	0.0221 (3)	0.0131 (2)	−0.00424 (19)	0.0019 (4)	−0.0007 (4)

### Geometric parameters (Å, º)

**Table:**

C1—N1	1.477 (4)	C6—H6C	0.9700
C1—H1C	0.9700	C6—H6B	0.9700
C1—H1B	0.9700	C6—H6A	0.9700
C1—H1A	0.9700	N1—Ni	2.1453 (19)
C4—N2	1.483 (3)	N2—Ni	2.141 (2)
C4—C3	1.511 (4)	Ni—O6	2.0639 (17)
C4—H4B	0.9800	Ni—O5	2.084 (2)
C4—H4A	0.9800	Ni—O1^i^	2.088 (2)
C2—N1	1.475 (4)	Ni—O2	2.0931 (19)
C2—H2C	0.9700	O1—S	1.471 (2)
C2—H2B	0.9700	O1—Ni^ii^	2.088 (2)
C2—H2A	0.9700	O2—S	1.4751 (19)
C3—N1	1.478 (3)	O3—S	1.4828 (18)
C3—H3B	0.9800	O4—S	1.472 (2)
C3—H3A	0.9800	O5—H1	0.834 (18)
C5—N2	1.484 (3)	O5—H2	0.841 (18)
C5—H5C	0.9700	O6—H3	0.825 (17)
C5—H5B	0.9700	O6—H4	0.830 (17)
C5—H5A	0.9700	O7—H5	0.832 (18)
C6—N2	1.477 (4)	O7—H6	0.829 (18)
			
N1—C1—H1C	109.5	C2—N1—Ni	112.29 (17)
N1—C1—H1B	109.5	C1—N1—Ni	112.36 (18)
H1C—C1—H1B	109.5	C3—N1—Ni	105.19 (13)
N1—C1—H1A	109.5	C6—N2—C4	109.5 (2)
H1C—C1—H1A	109.5	C6—N2—C5	107.6 (2)
H1B—C1—H1A	109.5	C4—N2—C5	108.4 (2)
N2—C4—C3	110.4 (2)	C6—N2—Ni	114.27 (17)
N2—C4—H4B	109.6	C4—N2—Ni	103.46 (15)
C3—C4—H4B	109.6	C5—N2—Ni	113.46 (16)
N2—C4—H4A	109.6	O6—Ni—O5	93.42 (8)
C3—C4—H4A	109.6	O6—Ni—O1^i^	88.42 (8)
H4B—C4—H4A	108.1	O5—Ni—O1^i^	89.20 (7)
N1—C2—H2C	109.5	O6—Ni—O2	88.97 (8)
N1—C2—H2B	109.5	O5—Ni—O2	88.25 (8)
H2C—C2—H2B	109.5	O1^i^—Ni—O2	176.24 (7)
N1—C2—H2A	109.5	O6—Ni—N2	90.77 (8)
H2C—C2—H2A	109.5	O5—Ni—N2	175.58 (8)
H2B—C2—H2A	109.5	O1^i^—Ni—N2	89.51 (8)
N1—C3—C4	110.7 (2)	O2—Ni—N2	93.23 (8)
N1—C3—H3B	109.5	O6—Ni—N1	175.51 (8)
C4—C3—H3B	109.5	O5—Ni—N1	91.06 (8)
N1—C3—H3A	109.5	O1^i^—Ni—N1	91.40 (9)
C4—C3—H3A	109.5	O2—Ni—N1	91.41 (8)
H3B—C3—H3A	108.1	N2—Ni—N1	84.74 (8)
N2—C5—H5C	109.5	S—O1—Ni^ii^	136.26 (12)
N2—C5—H5B	109.5	S—O2—Ni	138.52 (11)
H5C—C5—H5B	109.5	Ni—O5—H1	111 (2)
N2—C5—H5A	109.5	Ni—O5—H2	100 (2)
H5C—C5—H5A	109.5	H1—O5—H2	105 (3)
H5B—C5—H5A	109.5	Ni—O6—H3	117.8 (19)
N2—C6—H6C	109.5	Ni—O6—H4	108 (2)
N2—C6—H6B	109.5	H3—O6—H4	108 (3)
H6C—C6—H6B	109.5	H5—O7—H6	95 (3)
N2—C6—H6A	109.5	O1—S—O4	110.72 (12)
H6C—C6—H6A	109.5	O1—S—O2	108.00 (10)
H6B—C6—H6A	109.5	O4—S—O2	109.83 (12)
C2—N1—C1	107.7 (2)	O1—S—O3	109.19 (12)
C2—N1—C3	110.0 (2)	O4—S—O3	109.28 (11)
C1—N1—C3	109.3 (2)	O2—S—O3	109.80 (11)
			
N2—C4—C3—N1	58.5 (3)	C3—N1—Ni—O5	−171.47 (18)
C4—C3—N1—C2	−158.6 (2)	C2—N1—Ni—O1^i^	−141.1 (2)
C4—C3—N1—C1	83.4 (2)	C1—N1—Ni—O1^i^	−19.51 (19)
C4—C3—N1—Ni	−37.4 (3)	C3—N1—Ni—O1^i^	99.30 (18)
C3—C4—N2—C6	77.5 (3)	C2—N1—Ni—O2	36.4 (2)
C3—C4—N2—C5	−165.5 (2)	C1—N1—Ni—O2	158.00 (19)
C3—C4—N2—Ni	−44.8 (2)	C3—N1—Ni—O2	−83.19 (19)
C6—N2—Ni—O6	79.76 (18)	C2—N1—Ni—N2	129.6 (2)
C4—N2—Ni—O6	−161.23 (16)	C1—N1—Ni—N2	−108.89 (19)
C5—N2—Ni—O6	−44.05 (19)	C3—N1—Ni—N2	9.93 (18)
C6—N2—Ni—O1^i^	168.18 (18)	O6—Ni—O2—S	0.8 (2)
C4—N2—Ni—O1^i^	−72.82 (15)	O5—Ni—O2—S	−92.6 (2)
C5—N2—Ni—O1^i^	44.37 (19)	N2—Ni—O2—S	91.5 (2)
C6—N2—Ni—O2	−9.25 (18)	N1—Ni—O2—S	176.3 (2)
C4—N2—Ni—O2	109.75 (15)	Ni^ii^—O1—S—O4	−18.4 (2)
C5—N2—Ni—O2	−133.06 (19)	Ni^ii^—O1—S—O2	−138.68 (15)
C6—N2—Ni—N1	−100.37 (18)	Ni^ii^—O1—S—O3	102.0 (2)
C4—N2—Ni—N1	18.63 (15)	Ni—O2—S—O1	−135.26 (17)
C5—N2—Ni—N1	135.8 (2)	Ni—O2—S—O4	103.9 (2)
C2—N1—Ni—O5	−51.8 (2)	Ni—O2—S—O3	−16.3 (2)
C1—N1—Ni—O5	69.72 (19)		

Symmetry codes: (i) *x*, *y*, *z*−1; (ii) *x*, *y*, *z*+1.

### Hydrogen-bond geometry (Å, º)

**Table:**

*D*—H···*A*	*D*—H	H···*A*	*D*···*A*	*D*—H···*A*
O5—H2···O4^i^	0.84 (2)	1.84 (2)	2.672 (2)	171 (3)
O5—H1···O7	0.83 (2)	1.93 (2)	2.765 (3)	176 (3)
O6—H4···O3	0.83 (2)	1.89 (2)	2.687 (3)	160 (3)
O6—H3···O3^iii^	0.83 (2)	2.03 (2)	2.829 (3)	162 (3)
O7—H5···O4	0.83 (2)	2.12 (2)	2.899 (3)	157 (3)
O7—H6···O3^iii^	0.83 (2)	2.18 (2)	2.934 (3)	151 (3)

Symmetry codes: (i) *x*, *y*, *z*−1; (iii) −*x*, −*y*+1, *z*−1/2.
